# Space‐Time Wave Packets from Smith‐Purcell Radiation

**DOI:** 10.1002/advs.202100925

**Published:** 2021-10-17

**Authors:** Yi Ji Tan, Prakash Pitchappa, Nan Wang, Ranjan Singh, Liang Jie Wong

**Affiliations:** ^1^ Institute of Microelectronics Agency for Science, Technology and Research (A*STAR) 2 Fusionopolis Way Singapore 138634 Singapore; ^2^ School of Physical and Mathematical Sciences Nanyang Technological University 21 Nanyang Link Singapore 637371 Singapore; ^3^ School of Electrical and Electronic Engineering Nanyang Technological University 50 Nanyang Avenue Singapore 639798 Singapore

**Keywords:** electron bunches, free electron radiation, nanophotonic light sources, optical waveshaping, space‐time wave packets, terahertz, X‐ray

## Abstract

Space‐time wave packets are electromagnetic waves with strong correlations between their spatial and temporal degrees of freedom. These wave packets have gained much attention for fundamental properties like propagation invariance and user‐designed group velocities, and for potential applications like optical microscopy, micromanipulation, and laser micromachining. Here, free‐electron radiation is presented as a natural and versatile source of space‐time wave packets that are ultra‐broadband and highly tunable in frequency. For instance, ab initio theory and numerical simulations show that the intensity profile of space‐time wave packets from Smith‐Purcell radiation can be directly tailored through the grating properties, as well as the velocity and shape of the electron bunches. The result of this work indicates a viable way of generating space‐time wave packets at exotic frequencies such as the terahertz and X‐ray regimes, potentially paving the way toward new methods of shaping electromagnetic wave packets through free‐electron radiation.

## Graphical abstract

The intensity profile of a space‐time wave packet from Smith‐Purcell radiation resulting from the interaction between an electron and a grating. This space‐time wave packet consists of only the 2nd and 3rd order Smith‐Purcell radiation, achieved by controlling the electron's velocity and by applying a transmission frequency filter of desired bandwidth.

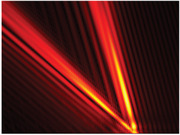

## 1 Introduction

Space‐time wave packets have attracted much attention due to their ability to propagate at arbitrary group velocities while maintaining an invariant electromagnetic intensity profile.^[^ ^1^, ^2^, ^3^, ^4^, ^5^, ^6^, ^7^, ^8^, ^9^, ^10^, ^11^, ^12^, ^13^, ^14^, ^15^, ^16^, ^17^, ^18^, ^19^ ^]^ Many such wave packets have been theoretically studied and experimentally realized, including accelerating Airy beams,^[^ ^20^, ^21^, ^22^ ^]^ tilted‐pulse‐front pulses,^[^ ^23^, ^24^, ^25^ ^]^ and abruptly focusing wave packets.^[^ ^26^, ^27^, ^28^, ^29^, ^30^ ^]^ Space‐time wave packets have potential applications in optical microscopy,^[^ ^31^, ^32^, ^33^, ^34^, ^35^ ^]^ micromanipulation of particles,^[^ ^36^, ^37^, ^38^ ^]^ and laser micromachining.^[^ ^39^, ^40^ ^]^ To date, realization of space‐time wave packets has been restricted to the use of light shaping elements. Here, we show that free‐electron radiation is a natural and versatile source of propagation‐invariant space‐time wave packets. We use Smith‐Purcell radiation as an example,^[^ ^41^ ^]^ showing that the scattering of an electron's evanescent fields off a grating is a robust platform for generating space‐time wave packets that are highly tunable in frequency from terahertz to soft X‐rays. For instance, our ab initio simulations show that an ultraviolet space‐time wave packet of central wavelength 193 nm, bandwidth 15 nm and peak intensity 56 MWm^–2^ can be generated from realistic 200 keV electron bunches interacting with a single‐layer amorphous silicon grating of period 100 nm. Our findings reveal that free‐electron radiation is a highly versatile platform for generating space‐time wave packets with tailored wave profiles and group velocities. Specifically, the space‐time wave properties are readily tuned through the electron velocity, grating geometry, grating material and frequency filter bandwidth. We show that the space‐time wave profile can be further manipulated by shaping the electron bunch using existing nanotip emitters,^[^ ^42^, ^43^, ^44^, ^45^, ^46^, ^47^, ^48^, ^49^, ^50^ ^]^ for instance, to control the position of the intensity hotspot. Our findings highlight the fundamental relationship between free‐electron radiation and space‐time wave packets, with potential applications in areas like ultrafast electron diffraction,^[^ ^51^, ^52^, ^53^ ^]^ laser‐driven electron acceleration,^[^ ^54^, ^55^, ^56^, ^57^, ^58^ ^]^ and Thomson scattering‐based X‐ray sources.^[^ ^59^, ^60^, ^61^ ^]^

## 2 Results

### 2.1 Ultraviolet Space‐Time Wave Packets from Smith‐Purcell Radiation

**Figure 1:**
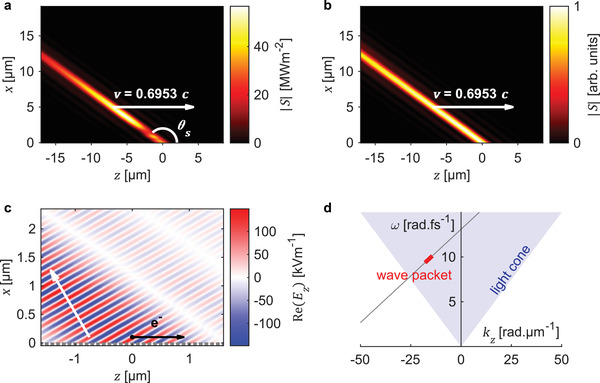
Ultraviolet space‐time wave packet from Smith‐Purcell radiation. a) Intensity profile of a space‐time wave packet (central wavelength 193 nm, bandwidth 15 nm) from Smith‐Purcell radiation, generated from an array of 200 keV electrons (total charge of 0.64 fC) interacting with a single‐layer amorphous silicon grating of period 100 nm. b) A space‐time wave packet with the same space‐time correlation as in (a), obtained by adding plane waves of equal amplitude and phase. c) Electric field profile of the space‐time wave packet in (a), showing the grating structure, electron position, and the direction of wavefront propagation (white arrow). d) Dispersion relation of the 1st order diffracted waves (gray) of the Smith‐Purcell radiation, which compose the space‐time wave packet (red).

**Figure** **1** shows an ultraviolet space‐time wave packet of central wavelength 193 nm, which results from Smith‐Purcell radiation after applying a transmission filter of bandwidth 15 nm.^[^ ^62^, ^63^, ^64^, ^65^ ^]^ Smith‐Purcell radiation is generated from an array of 200 keV electrons interacting with a single‐layer amorphous silicon grating of period 100 nm (Figure 1c).^[^ ^66^ ^]^ Our results are obtained through ab initio frequency domain simulations that capture the electromagnetic response of an electron's evanescent field scattering off a periodic grating (see Experimental Section).

The intensity profile of the space‐time wave packet in Figure 1a bears a striking resemblance to a space‐time wave packet produced by adding plane waves of equal amplitude and phase (Figure 1b). The wave packet in Figure 1b may be obtained, for instance, by using optical elements such as spatial light modulators.^[^ ^67^, ^68^, ^69^ ^]^ This similarity highlights the potential of Smith‐Purcell radiation as a platform for generating space‐time wave packets. While there have been many studies on Smith‐Purcell radiation,^[^ ^70^, ^71^, ^72^, ^73^, ^74^ ^]^ none of them have explored this free‐electron radiation mechanism as a platform for space‐time wave packet generation. The inherent space‐time correlation in Smith‐Purcell radiation can in fact be directly seen from the relationship between temporal frequency (*ω*) and spatial frequency (*k_zm_*), as illustrated in Figure 1d: *k*_zm_ = *ω*/*v* + 2*πm*/*d*, where *k*_zm_ is the *z*‐directed wave‐vector component of diffraction order *m*, *v* is the electron's velocity (also the group velocity of the resulting space‐time wave packet) and *d* is the grating period. We see that the frequency and directionality of the space‐time wave packet can be tuned by controlling the electron velocity, grating period, and frequency filter bandwidth. The phase velocity of the space‐time wave packet (along the electron's propagation direction) is given by *v*_ph_ = *ω*/*k*_zm_, which can also be tuned by varying the electron velocity and grating period. Controlling the filtered frequencies to include only the 1st order Smith‐Purcell radiation, for instance, leads to the intensity profile shown in Figure 1a. We also obtain the angle *θ*_s_ (shown in Figure 1a), given by

(1) $$\cos\left(\pi -\theta_{s}\right)=\left(\beta -\cos\theta_{m}\right)\;{\left(1+\beta^{2}-2\beta \cos\theta_{m}\right)}^{-1/2}$$

where normalized velocity *β = v/c*, propagation angle $\theta_{m}=\arccos\left(k_{\mathrm{zm}}/k\right)$, wave‐vector *k = ω/c, and c* is the speed of light in vacuum. The intensity profile can also be tailored through the electron bunch shape. For instance, the 56 MWm^–2^ intensity hotspot in Figure 1a, which is absent in Figure 1b, results from using an array of 200 keV electrons (total charge of 0.64 fC) equally spaced across 1.6 µm along the *y*‐direction, positioned 100 nm above the grating surface. This electron configuration is shown in Figure 1c, which is an array of point charges that lies along the *y*‐direction. We later show that the intensity profile of space‐time wave packets remains relatively unchanged even when we consider an array of electron bunches, where the electrons are normally distributed (Gaussian distribution). We refer to the full‐width‐at‐half‐maximum diameter of the electron distribution as spot size, and we consider in this study spot sizes that are smaller than the desired emission wavelengths. Such electron bunches can be obtained from existing electron sources,^[^ ^75^, ^76^ ^]^ which have been used to demonstrate the generation of 120 keV electron bunches with 0.655 fs pulse duration, and 70 keV electron bunches with 0.82 fs pulse duration.

### 2.2 Versatile Space‐Time Wave Packet Design from Frequency Filtering

**Figure 2:**
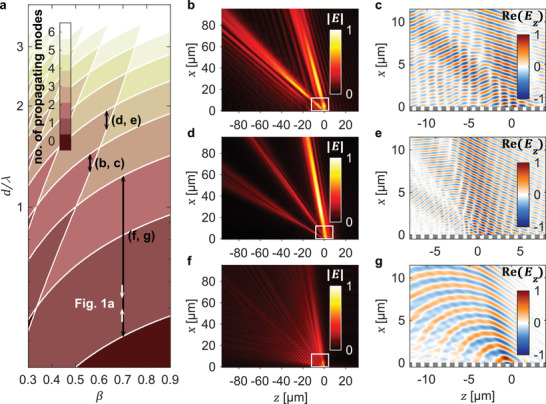
Versatile space‐time wave profiles. a) Colormap representing the number of propagating diffraction modes in Smith‐Purcell radiation as a function of normalized velocity (*β* = *v*/*c*) and grating period to wavelength ratio (*d*/*λ*). The vertical arrows indicate the filtered frequency range for the space‐time wave packets shown in Figure 1a and the subfigures (b–g). The electric field profile within the region of the white box in (b), (d), and (f) are shown in (c), (e), and (g), respectively, along with the grating structure. The space‐time wave packets are generated from an array of electrons (equally spaced across 16 µm along the *y*‐direction) interacting with a grating (*ε*_r_ = 2.25) of period 1 µm, for an electron velocity and filter bandwidth of *β* = 0.56, Δ*λ* = 89.3 nm in (b, c), *β* = 0.63, Δ*λ* = 69.8 nm in (d, e), and *β* = 0.7, Δ*λ* = 1.6 µm in (f, g). Our results show how the space‐time wave profile can be shaped simply by adjusting the filtered frequency range and bandwidth.

In **Figure** **2**, we show space‐time wave packets of different field profiles that can be generated from Smith‐Purcell radiation. We use a grating (relative permittivity *ε*_r_ = 2.25) of period 1 µm to realize the different field profiles by applying different frequency filters. Since Smith‐Purcell radiation is an ensemble of diffracted waves containing arbitrarily high diffraction orders, a wide range of space‐time wave patterns can be created simply by varying the filter bandwidth, which in turn controls the number of diffraction orders in the designed space‐time wave packet.

Figure 2a shows the number of diffraction orders present in the space‐time wave packet as a function of normalized velocity (*β* = *v*/*c*) and grating period to wavelength ratio (*d*/*λ*). The vertical arrows indicate the filtered frequency range of the space‐time wave packets shown in Figure 2b,d,f, and their vertical orientation enforces the constant group velocity of the overall space‐time wave packet. Figure 2b,c shows the normalized electric field profile of a space‐time wave packet that comprises only the 2nd and 3rd order Smith‐Purcell radiation. This is realized by applying a frequency filter centered at 741 nm wavelength with 89.3 nm bandwidth. Similarly, a space‐time wave packet that consists of only the 2nd, 3rd, and 4th order Smith‐Purcell radiation is depicted in Figure 2d,e, obtained using a frequency filter centered at 552 nm wavelength with 69.8 nm bandwidth. Figure 2f,g shows a space‐time wave packet of central wavelength 1.6 µm and a much broader bandwidth of 1.6 µm. We thus see that a variety of space‐time wave packets can be designed via Smith‐Purcell radiation by varying the filter bandwidth, opening up a wide range of intriguing space‐time wave packet designs, limited only by the frequencies and bandwidths available in existing frequency filters.

### 2.3 Space‐Time Wave Packets from the Terahertz to X‐ray Regimes

**Figure 3:**
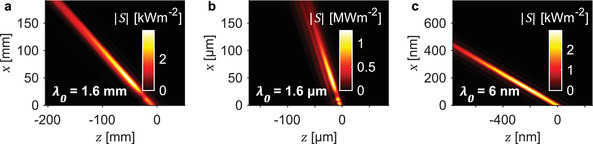
Frequency tunable space‐time wave packets from terahertz to X‐ray regimes. Intensity profile of space‐time wave packets in the (a) terahertz, (b) infrared, and (c) soft X‐ray regimes. a) A terahertz space‐time wave packet (central frequency 0.187 THz, bandwidth 20 GHz) is generated from 20 pC, 200 keV electron bunches interacting with a silicon grating (*ε*_r_ = 11.7) of period 1 mm. b) An infrared space‐time wave packet (central wavelength 1.6 µm, bandwidth 170 nm) is generated from 0.2 fC, 100 keV electron bunches interacting with a silicon grating (*ε*_r_ = 11.7) of period 1 µm. c) A soft X‐ray space‐time wave packet (central wavelength 6 nm, bandwidth 0.6 nm) is generated from two 0.2 fC, 5 MeV electron bunch interacting with a grating (*ε*_r_ ≈ 0.99) of period 4 nm. Our results show the high frequency tunability of space‐time wave packets generated by free‐electron radiation. In particular, Smith‐Purcell radiation can be used to generate space‐time wave packets in extreme frequency regimes where wave‐shaping elements are less accessible.

The peak frequency of space‐time wave packets from Smith‐Purcell radiation can be controlled by varying the period of the grating. Smith‐Purcell radiation has already been demonstrated at frequencies ranging from the terahertz to ultraviolet regimes.^[^ ^77^, ^78^, ^79^, ^80^ ^]^ We show here that this high tunability can be leveraged to generate tunable space‐time wave packets in exotic frequency regimes at which spatial light modulators, and other optical components typically used for space‐time wave packet generation, are not readily available. In **Figure** **3**, we show the intensity profile of space‐time wave packets from Smith‐Purcell radiation at terahertz, infrared, and soft X‐ray frequencies, generated using realistic grating and electron bunch geometries. Each electron bunch is modeled as a Gaussian distribution with a full‐width‐at‐half‐maximum diameter that we denote as spot size throughout this work.

Figure 3a shows the intensity profile of a terahertz space‐time wave packet (central frequency 0.187 THz, bandwidth 20 GHz) generated from 20 pC, 200 keV electron bunches (0.5 mm spot size and 2.4 ps pulse duration) which are obtainable from existing field emitter arrays.^[^ ^81^, ^82^, ^83^ ^]^ By using 16 of such electron bunches equally spaced across 16 mm along the *y*‐direction, centered 1 mm above the surface of a 1 mm period silicon grating (*ε*_r_ = 11.7), we obtain a 3.82 kWm^–2^ intensity hotspot located a few centimeters away from the grating surface. In Figure 3b, we show an infrared space‐time wave packet (central wavelength 1.6 µm, bandwidth 170 nm) generated from 0.2 fC, 100 keV electron bunches of 0.25 µm spot size and 1.5 fs pulse duration. These femtosecond electron pulses can be obtained from existing nanotip emitters.^[^ ^42^, ^43^, ^44^, ^45^ ^]^ Using 16 of such electron bunches equally spaced across 16 µm along the *y*‐direction, centered 0.5 µm above the surface of a 1 µm period silicon grating (*ε*_r_ = 11.7), we achieve a 1.27 MWm^–2^ intensity hotspot located about 50 µm away from the grating surface. The intensity of these space‐time wave packets can be further enhanced by using relativistic electrons, which can be obtained by accelerating electrons up to energies of 5 MeV using radiofrequency cavities.^[^ ^84^ ^]^ Using relativistic electrons also reduces the bunch divergence due to electromagnetic repulsion between electrons, allowing the electron bunch to remain focused over a longer distance. Figure 3c shows a soft X‐ray space‐time wave packet (central wavelength 6 nm, bandwidth 0.6 nm) of peak intensity 2.94 kWm^–2^, generated from two 0.2 fC, 5 MeV electron bunch (50 nm spot size and 0.02 fs pulse duration) interacting with a 4 nm period grating (*ε*_r_ ≈ 0.99). Such attosecond electron bunches can be realized via bunch compression using ultrashort laser pulses.^[^ ^85^, ^86^, ^87^, ^88^ ^]^

### 2.4 Tailoring the Intensity Profile of Space‐Time Wave Packets by Electron Bunch Shaping

**Figure 4:**
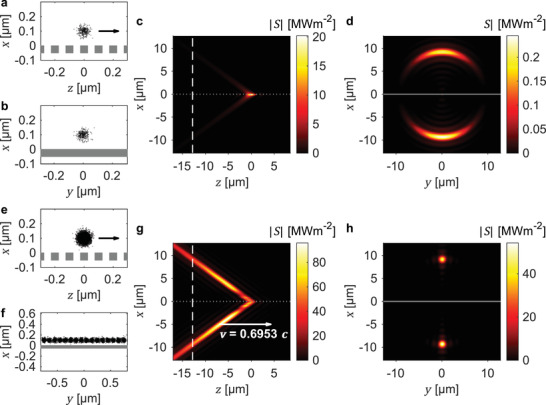
Tailoring the intensity profile of space‐time wave packets by electron bunch shaping. a,b) Normally distributed 0.04 fC, 200 keV electron bunch of 50 nm spot size, centered 100 nm above the surface of a 100 nm period amorphous silicon grating. c,d) Intensity profile of a space‐time wave packet (central wavelength 193 nm, bandwidth 15 nm) generated from the electron bunch distribution in (a, b). e,f) An array of 16 electron bunches spanning across 1.6 µm along the *y*‐direction. g,h) Intensity profile of a space‐time wave packet generated from the electron bunch distribution in (e, f), showing a beam‐like intensity profile with a 96 MWm^–2^ intensity hotspot (1.3 µm transverse spot size) located a few micrometers away from the grating surface. Our results show that the intensity profile of space‐time wave packets from free‐electron radiation can be shaped—e.g., to become more focused transversely in this case—by controlling the electron bunch distribution.

In **Figure** **4**, we show that the intensity profile of space‐time wave packets from Smith‐Purcell radiation can be controlled by shaping the spatial distribution of the incident electron bunches. We consider an ultraviolet space‐time wave packet of central wavelength 193 nm and bandwidth 15 nm generated from 0.04 fC, 200 keV electron bunches (50 nm spot size and 0.24 fs pulse duration) interacting with a single‐layer amorphous silicon grating of period 100 nm. A single electron bunch (Figure 4a,b) produces a space‐time wave packet with an intensity hotspot located at the grating surface (Figure 4c) that radiates with a conical‐like intensity profile (Figure 4d). By using an array of electron bunches equally spaced along the *y*‐direction (Figure 4e,f), this intensity hotspot is refocused to a position away from the grating surface (Figure 4g). In addition to the relocation of the intensity hotspot, a narrow transverse hotspot of 1.3 µm spot size is also achieved (Figure 4h), which results from the interference of radiation from the electron bunch array. Furthermore, the ability to relocate the intensity hotspot away from the grating surface allows the hotspot to be arbitrarily positioned, making it more flexible to implement in potential applications. We also note that a second space‐time wave packet is produced on the other side of the grating (Figure 4g,h). By applying different frequency filters on either side of the grating, more than one space‐time wave packet can be simultaneously generated.

## 3 Discussion

Our observation that free‐electron radiation is a natural, versatile source of space‐time wave packets is fundamentally intriguing, as it indicates that strong space‐time correlations in electromagnetic radiation is much more ubiquitous than commonly suspected. As a method for generating space‐time wave packets, free‐electron radiation is complementary to existing methods that use optical elements, by filling the technological gap at extreme frequency regimes—such as extreme ultraviolet and X‐rays—where optical elements typically used for wave‐shaping are not as readily available. Broadband space‐time wave packets are naturally derived from free‐electron radiation by applying broadband frequency filters. Such broadband filters have been demonstrated at frequencies from the terahertz to ultraviolet regimes,^[^ ^63^, ^64^, ^89^, ^90^, ^91^, ^92^, ^93^, ^94^ ^]^ including terahertz filters with 0.5 THz bandwidth,^[^ ^95^ ^]^ infrared filters centered at 1650 nm wavelength with 225 nm bandwidth,^[^ ^96^ ^]^ and ultraviolet filters with central wavelength ranging from 180 to 240 nm and bandwidths up to 31 nm.^[^ ^62^ ^]^

The intensity profile of space‐time wave packets from Smith‐Purcell radiation can be tuned not only through the frequency filter, but also through the electron energy, grating geometry, and electron bunch shape. Electron bunch shaping can be achieved through techniques that control both the longitudinal and transverse electron bunch profile using magnetic elements and light pulses.^[^ ^97^, ^98^, ^99^ ^]^ The intensity of space‐time wave packets at terahertz frequencies can be further enhanced by using high‐density field emitter arrays capable of emitting 200 pC electron bunches with 1 mm spot size and 5 ps pulse duration.^[^ ^81^, ^82^, ^83^, ^100^ ^]^ At X‐ray frequencies, space‐time wave packets can also be generated from Smith‐Purcell radiation using relativistic electron bunches with nanometer spot size and attoseconds pulse duration,^[^ ^85^, ^86^, ^87^, ^88^ ^]^ limited only by the availability of transmission filters at such short wavelength regimes.

Although our study considers only the case of a single‐layer grating, our ab initio simulation tool can be employed to calculate Smith‐Purcell radiation from an arbitrary multilayer grating structure (see Experimental Section). Multilayer grating structures have been fabricated and used to enhance diffraction efficiencies at the infrared,^[^ ^101^, ^102^ ^]^ optical,^[^ ^103^ ^]^ and X‐ray frequency regimes.^[^ ^104^, ^105^ ^]^ While the group velocity of space‐time wave packet is unaffected by the grating geometry and material, the spectral distribution of Smith‐Purcell radiation can be controlled using multiple grating layers of different materials, periodicities, and thicknesses. For instance, the intensity of Smith‐Purcell radiation can be enhanced using dielectric loaded gratings,^[^ ^106^, ^107^, ^108^ ^]^ and Smith‐Purcell radiation can be simultaneously generated at multiple frequencies (for a given propagation direction) using a super grating structure with two grating layers of different periodicity.^[^ ^109^ ^]^ Hence, the design of multilayer gratings offers new ways of controlling the field profiles of space‐time wave packets generated by Smith‐Purcell radiation.

While we have only considered Smith‐Purcell radiation in our study, other forms of radiation may also be present through the collision of stray electrons with the grating material. This leads to sources of background noise through inelastic scattering processes such as transition radiation and Bremsstrahlung.^[^ ^110^ ^]^ These undesired contributions can be reduced by positioning the electron bunches farther from the grating surface, which would still allow space‐time wave packets to be generated via Smith‐Purcell radiation, albeit at lower intensities. High quality, well collimated electron beams are therefore desirable in our application, as they allow greater proximity to the grating surface while minimizing background radiation produced by stray electrons.

Although we have focused on Smith‐Purcell radiation in this study, we note that the fundamental connection between Smith‐Purcell radiation and space‐time wave packets arises from the periodic nature of the diffracting medium and the uniform motion of the free electron. This implies that other free‐electron radiation mechanisms can also be leveraged to generate space‐time wave packets. These radiation mechanisms include, but are not limited to, Cherenkov radiation,^[^ ^111^, ^112^ ^]^ for which threshold‐less Cherenkov radiation emitters have been demonstrated using hyperbolic metamaterials,^[^ ^113^ ^]^ and diffractive radiation from periodic light well structures.^[^ ^114^, ^115^ ^]^ Space‐time wave packets are thus much more ubiquitous in nature and in the laboratory than is commonly thought.

Given the subluminal nature of all massive particles, we note that only space‐time wave packets with subluminal group velocities can be generated from free electrons. Subluminal space‐time wave packets have potential applications in ultrafast electron diffraction ^[^ ^51^, ^52^, ^53^ ^]^ and Thomson scattering‐based X‐ray sources,^[^ ^59^, ^60^, ^61^ ^]^ where group velocity matching between light and electrons can lead to extended interaction times and stronger coupling. Group velocity matching is also crucial in laser‐driven electron acceleration schemes such as wakefield acceleration and ponderomotive acceleration.^[^ ^54^, ^56^, ^116^, ^117^ ^]^ Group velocity matching can be important in direct acceleration schemes,^[^ ^54^, ^55^, ^57^, ^118^, ^119^, ^120^, ^121^, ^122^, ^123^ ^]^ where phase velocity matching is also crucial, especially when ultrashort laser pulses are involved.^[^ ^54^, ^55^, ^57^ ^]^

We note that space‐time wave packets from free‐electron radiation are propagation‐invariant only as far as space charge effects remains negligible. This allows for reasonably long propagation distances for a wide range of electron bunch radii and energies (see Supporting Information). For example, our calculations show that a 0.04 fC, 200 keV electron bunch (50 nm spot size and 0.24 fs pulse duration) remains relatively propagation‐invariant over a distance of 10 µm (>1600 times the wavelength of a soft X‐ray space‐time wave packet of central wavelength 6 nm), with a maximum gain of 0.16% energy spread. Longer propagation distances can be realized by decreasing the density of electrons within each electron bunch, and by using relativistic electrons of higher energy.

So far, we have considered free electrons as classical point charges, whose emitted photons interfere with one another to form a space‐time wave packet. It should be noted, however, that different output photon states are entangled with different outgoing electron states even when a single input electron is considered. As such, photons of different frequencies or angles interfere to form a coherent wave packet, only if these photons are spectrally coherent.^[^ ^124^ ^]^ The degree of spectral coherence between these output photons has been shown to be dependent on the coherence of the emitting free electron.^[^ ^125^ ^]^ In particular, the required coherent energy uncertainty of the input electron should be at least comparable with the energy bandwidth of the output photon wave packet. This is less challenging at microwave and terahertz frequencies, where a relatively low coherent energy uncertainty (on the order of the photon energy) is needed to span the entire bandwidth of the emitted photon wave packet. Nevertheless, electron wave packets of larger coherent energy uncertainties are also feasible with modern technology. This can be inferred from experimental realizations of electron pulse durations demonstrating coherent energy uncertainties on the order of 1 eV or higher,^[^ ^75^, ^126^, ^127^ ^]^ as well as theoretical predictions of attosecond electron pulses associated with coherent energy uncertainties up to hundreds of electron volts.^[^ ^127^, ^128^, ^129^ ^]^ In comparison, our ultraviolet space‐time wave packet in Figure 1a (generated using 200 keV electrons) has an energy spread of 0.5 eV, while the wave packets in Figure 3 have photon energy spreads of 82.7 μeV and 83.3 meV for the terahertz and infrared examples, respectively. These space‐time wave packets can thus be realized with existing electron sources. Importantly, it should be noted that even with arbitrarily limited coherent energy uncertainty in the input electrons, the fundamental phenomena we predict—namely the generation of space‐time wave packets from Smith‐Purcell radiation—can take place at any frequency regime. The main caveat is that with limited coherent energy uncertainty in the input electrons, the coherent bandwidth of the resulting space‐time wave packets would be smaller, and the resulting intensity hotspot would have a larger width.

## 4 Conclusion

We have presented Smith‐Purcell radiation as a promising platform for generating broadband space‐time wave packets that are highly tunable in frequency and wave profile. We showed that intense, broadband ultraviolet space‐time wave packet of peak intensity 56 MWm^–2^ can be generated from 0.04 fC, 200 keV electron bunches that are available from existing nanotip emitters. We showed that free‐electron radiation is a source of space‐time wave packets in extreme frequency regimes where the required optical shaping elements are not as readily available, such as terahertz and soft X‐rays. Specifically, our ab initio simulations predict the generation of terahertz and soft X‐ray space‐time wave packets, with peak intensities 3.82 and 2.94 kWm^–2^, from 200 keV and 5 MeV electron bunches, respectively. Such electron bunches are available from existing field emitter arrays and attosecond electron sources. Beyond Smith‐Purcell radiation, our findings indicate that other free‐electron radiation mechanisms are potential sources of space‐time wave packets. These space‐time wave packets have potential applications in laser‐driven particle acceleration, ultrafast electron diffraction, and compact X‐ray generation schemes, where the group velocity matching between particles and light pulses are important for maximizing their interaction time. Our findings should pave the way to a wider range of techniques for shaping light with space‐time correlations.

## 5 Experimental Section

The ab initio simulations performed in this work solve the full Maxwell's equations in the frequency domain, in response to excitation by electrons. Specifically, the electromagnetic response of a periodic grating is considered as a superposition of diffracted waves due to the incident fields of an electron described by the Liénard–Wiechert potentials.^[^ ^130^, ^131^ ^]^

### Electromagnetic Fields of Diffracted Waves from a Grating

**Figure 5:**
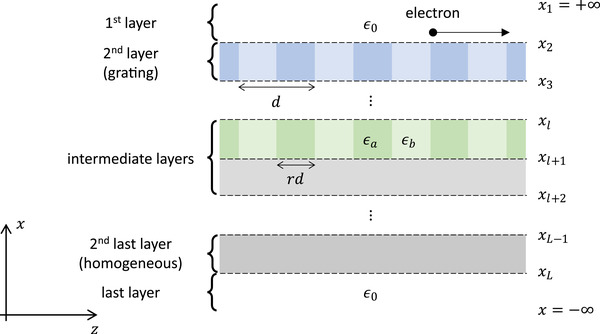
Homogeneous and grating layers of an arbitrary multilayer structure for ab initio Smith‐Purcell simulations. An arbitrary multilayer structure is separated into homogeneous and grating layers defined within the region *x*_l + 1_ ≤ *x* < *x*_l_, where *x*_l + 1_ and *x*_l_ are the lower and upper boundaries of the *l*th layer, respectively. Each grating layer is a periodic medium that consist of material *a* and *b*, defined within the region − *rd*/2 < *z* ≤ *rd*/2 and *rd*/2 < *z* ≤ (*d* − *rd*/2), with wave‐vector $k_{a}=\;\omega \sqrt{\varepsilon_{a}\mu_{a}}$ and $k_{b}=\;\omega \sqrt{\varepsilon_{b}\mu_{b}}$, respectively. The first and last layers are defined to be homogeneous, with the total electromagnetic fields described as a sum of diffracted waves.

The dispersion relation of waves diffracted by a grating (stratified along the *y*‐direction and periodic in the *z*‐direction) is given by the grating equation *k*_zm_ = *k*_zi_ + 2*πm*/*d*, where *k*_zm_ is the *z*‐directed wave‐vector component of diffraction order *m*, *k*_zi_ is the incident wave‐vector and *d* is the grating period. To formulate the diffraction of an incident electromagnetic field as a scattering problem, the spatial domain is separated along the *x*‐direction into homogeneous and grating layers defined within the region *x*_l + 1_ ≤ *x* < *x*_l_ as illustrated in **Figure** **5**, where *x*_l + 1_ and *x*_l_ are the lower and upper boundaries of the *l*th layer, respectively.

The diffracted waves in a homogeneous layer at frequency (*ω*) and transverse wave‐vector (*k*_y_) is described as a superposition of waves (which may be propagating or evanescent) with the *y*‐component of electromagnetic fields given by

(2a) $$E_{y}=\sum_{m=-\infty }^{\infty }\left(a_{m}e^{ik_{\mathrm{xm}}x}+b_{m}e^{-ik_{\mathrm{xm}}x}\right)e^{ik_{\mathrm{zm}}z}e^{i\left(k_{y}y-\omega t\right)}\;$$

(2b) $$H_{y}=\frac{1}{Z}\;\sum_{m\;=\;-\infty }^{\infty }\left(c_{m}e^{ik_{\mathrm{xm}}x}+d_{m}e^{-ik_{\mathrm{xm}}x}\right)e^{ik_{\mathrm{zm}}z}e^{i\left(k_{y}y-\omega t\right)}$$

where *a*_m_, *b*_m_, *c*_m_, and *d*_m_ are complex coefficients of the *m*th order diffracted waves with transverse wave‐vector $k_{\mathrm{xm}}={\left(k^{2}-k_{y}^{2}-k_{\mathrm{zm}}^{2}\right)}^{1/2}\;$, propagation wave‐vector $k\;=\;\omega \sqrt{\varepsilon \mu }$, and wave impedance $Z=\sqrt{\mu /\varepsilon }\;$ with electric permittivity *ε* and magnetic permeability *μ*. Whereas Equations (2a) and (2b) describe the electromagnetic waves in a homogenous layer, the electromagnetic modes in a grating layer is defined as a superposition of waves given by

(3a) $$E_{y}=\sum_{n}\left(A_{n}e^{ik_{\mathrm{zn}}z}+B_{n}e^{-ik_{\mathrm{zn}}z}\right)e^{ik_{\mathrm{xn}}x}e^{i\left(k_{y}y-\omega t\right)}$$

(3b) $$H_{y}=\frac{1}{Z}\;\sum_{n}\left(C_{n}e^{ik_{\mathrm{zn}}z}+D_{n}e^{-ik_{\mathrm{zn}}z}\right)e^{ik_{\mathrm{xn}}x}e^{i\left(k_{y}y-\omega t\right)}$$

where *A*_n_, *B*_n_, *C*_n_, and *D*_n_ are complex coefficients of the *n*th eigenmode with *z*‐directed wave‐vector component $k_{\mathrm{zn}}={\left(k^{2}-k_{y}^{2}-k_{\mathrm{xn}}^{2}\right)}^{1/2}$. The other Cartesian components of electric field are obtained from the Maxwell's equations as

(4a) $$E_{x}=\frac{1}{k^{2}-k_{y}^{2}}\;\left(ik_{y}\frac{\partial E_{y}}{\partial x}-i\omega \mu \frac{\partial H_{y}}{\partial z}\right)$$

(4b) $$E_{z}=\frac{1}{k^{2}-k_{y}^{2}}\;\left(ik_{y}\frac{\partial E_{y}}{\partial z}+i\omega \mu \frac{\partial H_{y}}{\partial x}\right)$$

The expressions for the magnetic fields *H*_x_ and *H*_z_ are then obtained from the electric fields by applying the exchange operation ***E*** ↔ ***H*** and + *ε*↔ − *μ*.

Whereas the diffracted wave‐vectors *k*_zm_ are known, the eigenmode wave‐vectors *k*_xn_ are determined from the electromagnetic interface and periodic boundary conditions within the grating layer. The derived electromagnetic field expressions allow the computation of the electromagnetic response of a multilayered structure (Figure 5), which may consist of an arbitrary number of homogeneous or grating layers, due to an incident electromagnetic field.

### Fourier Decomposition of the Electron's Electromagnetic Fields

Similarly, the electromagnetic fields of an electron is decomposed into frequency (*ω*) and wave‐vector (*k*_y_) components based on the definition of the magnetic vector potential of an arbitrarily moving point charge in free space, given by

(5) $$A\left(r,t\right)=\frac{\mu_{0}q}{{\left(2\pi \right)}^{4}}\;\;\int \int \int \;\frac{v\left(t^{\prime }\right)}{k^{2}-\omega^{2}/c^{2}}e^{ik\cdot \left(r-r_{0}\left(t^{\prime }\right)\right)}e^{-i\omega \left(t-t^{\prime }\right)}d^{3}k\;d\omega \;dt^{\prime }$$

where *μ*_0_ is the permeability of free space, *q* is the electron's charge, **v**(*t*′) and **r**_0_(*t*′) are the electron's velocity and position at retarded time *t*′, wave‐vector $k=k_{x}\;\hat{x}+k_{y}\hat{y}+k_{Z}\hat{z}$, and *c* is the speed of light in vacuum. Equation (5) is consistent with the Liénard–Wiechert potentials and can be derived from it. For a constant velocity $v\;=\;v\hat{z}$, evaluation of the integral in *k*_z_ followed by a contour integration in *k*_x_ results in the mathematical expression for the Fourier decomposition of the electron's magnetic vector potential, given by

(6) $$A\left(r,t\right)=\hat{z\;}\frac{v}{\left|v\right|}\frac{i\mu_{0}q}{8\pi^{2}}\;\int \;\;\;\;\int \frac{e^{ik_{x}\left|x-x_{0}\right|}}{k_{x}}e^{i\left(k_{y}y+k_{z}z-\omega t\right)}dk_{y}\;d\omega$$

where *x*‐directed wave‐vector component $k_{x}={\left(\omega^{2}/c^{2}-k_{y}^{2}-k_{z}^{2}\right)}^{1/2}\;$, and *k*_z_ = *ω*/*v*. By assuming the Lorenz gauge condition $\nabla \cdot A+\frac{1}{c^{2}}\;\frac{\partial \varphi }{\partial t}=\;0$, the electric potential (*φ*) is obtained from Equation (6), and the electromagnetic fields are determined from the electromagnetic potentials given by $E=-\nabla \varphi -\frac{\partial A}{\partial t}$ and $H\;=\frac{1}{\mu_{0}}\;\left(\nabla \times A\right)$. The electromagnetic fields of an electron are evanescent (imaginary *k*_x_) due to its subluminal velocity (*v* < *c*).

### Eigenmode Wave‐Vectors

The electromagnetic interface condition requires the tangential electromagnetic fields to be continuous across the interface between two materials. Each grating layer is a periodic medium with filling factor *r*, where material *a* is defined for the region − *rd*/2 < *z* ≤ *rd*/2 and material *b* for *rd*/2 < *z* ≤ (*d* − *rd*/2), with propagation wave‐vectors given by $k_{a}=\;\omega \sqrt{\varepsilon_{a}\mu_{a}}$ and $k_{b}=\;\omega \sqrt{\varepsilon_{b}\mu_{b}}$ respectively. Since the interface between material *a* and *b* lies in the *xy* plane, the electric fields *E*_x_ and *E*_y_ must be continuous. In the absence of a surface current, the magnetic fields *H*_x_ and *H*_y_ must be continuous as well. By excluding the phase factor $e^{i(k_{y}y-\omega t)}$, the tangential electromagnetic fields can be expressed in the matrix form as:

(7) $$\begin{array}{ccc} \left[\begin{array}{c} E_{\mathrm{yn}}\\ H_{\mathrm{xn}}\\ H_{\mathrm{yn}}\\ E_{\mathrm{xn}} \end{array}\right] & = & \Omega \left(\varepsilon ,\mu \right)\Lambda \left(k,k_{y}\right)K\left(k,k_{y},k_{x}\right)\Psi \left(k,k_{y},k_{x},\;z\right)\;\left[\begin{array}{c} A_{n}\\ B_{n}\\ C_{n}\\ D_{n} \end{array}\right]\\ & = & M\left(k,k_{y},k_{x},z\right)\left[\begin{array}{c} A_{n}\\ B_{n}\\ C_{n}\\ D_{n} \end{array}\right] \end{array}$$

where the matrices **Ω**(*ε*, *μ*), **Λ**(*k*, *k*_y_), $K(k,k_{y},k_{x})$ and **Ψ**(*k*, *k*_y_,*k*_x_, *z*) are defined as

(8a) $$\Omega \;\left(\varepsilon ,\mu \right)=\left[\begin{array}{cccc} 1 & 0 & 0 & 0\\ 0 & \frac{1}{Z} & 0 & 0\\ 0 & 0 & \frac{1}{Z} & 0\\ 0 & 0 & 0 & 1 \end{array}\right]\;$$

(8b) $$\Lambda \;\left(k,k_{y}\right)=\left[\begin{array}{cccc} 1 & 0 & 0 & 0\\ 0 & \frac{1}{k^{2}-k_{y}^{2}} & 0 & 0\\ 0 & 0 & 1 & 0\\ 0 & 0 & 0 & \frac{1}{k^{2}-k_{y}^{2}} \end{array}\right]$$

(8c) $$K\;\left(k,k_{y},k_{x}\right)=\left[\begin{array}{cccc} 1 & 1 & 0 & 0\\ -kk_{z} & +kk_{z} & -k_{y}k_{x} & -k_{y}k_{x}\\ 0 & 0 & 1 & 1\\ -k_{y}k_{x} & -k_{y}k_{x} & +kk_{z} & -kk_{z} \end{array}\right]$$

(8d) $$\Psi \;\left(k,k_{y},k_{x},\;z\right)=\left[\begin{array}{cccc} e^{ik_{z}z} & 0 & 0 & 0\\ 0 & e^{-ik_{z}z} & 0 & 0\\ 0 & 0 & e^{ik_{z}z} & 0\\ 0 & 0 & 0 & e^{-ik_{z}z} \end{array}\right]$$

with *z*‐directed wave‐vector component $k_{z}={\left(k^{2}-k_{y}^{2}-k_{x}^{2}\right)}^{1/2}\;$. The condition of field continuity between material *a* and *b* at *z* = *rd*/2 is given by

(9) $$M\left(k_{a},k_{y},k_{x},\frac{rd}{2}\right)\;\left[\begin{array}{c} A_{\mathrm{na}}\\ B_{\mathrm{na}}\\ C_{\mathrm{na}}\\ D_{\mathrm{na}} \end{array}\right]=\;M\left(k_{b},k_{y},k_{x},\frac{rd}{2}\right)\left[\begin{array}{c} A_{\mathrm{nb}}\\ B_{\mathrm{nb}}\\ C_{\mathrm{nb}}\\ D_{\mathrm{nb}} \end{array}\right]$$

where (*A*_na_,*B*_na_,*C*_na_,*D*_na_) and (*A*_nb_,*B*_nb_,*C*_nb_,*D*_nb_) are the complex coefficients of the *n*th eigenmode in region *a* and *b*, respectively. The periodic boundary condition for a grating that is infinitely periodic along the *z*‐direction is given by

(10) $$\begin{array}{c} e^{ik_{\mathrm{zi}}d}M\left(k_{a},k_{y},k_{x},-\frac{rd}{2}\right)\;\left[\begin{array}{c} A_{\mathrm{na}}\\ B_{\mathrm{na}}\\ C_{\mathrm{na}}\\ D_{\mathrm{na}} \end{array}\right]=M\left(k_{b},k_{y},k_{x},d-\frac{rd}{2}\right)\left[\begin{array}{c} A_{\mathrm{nb}}\\ B_{\mathrm{nb}}\\ C_{\mathrm{nb}}\\ D_{\mathrm{nb}} \end{array}\right] \end{array}$$

Combining the two conditions in Equations (9) and (10) gives the following eigenvalue equation:

(11) $$\begin{array}{c} \begin{array}{c} \left[e^{ik_{\mathrm{zi}}d}M\left(k_{a},-\frac{rd}{2}\right)-M\left(k_{b},d-\frac{rd}{2}\right)M^{-1}\left(k_{b},\frac{rd}{2}\right)M\left(k_{a},\frac{rd}{2}\right)\right]\;\left[\begin{array}{c} A_{\mathrm{na}}\\ B_{\mathrm{na}}\\ C_{\mathrm{na}}\\ D_{\mathrm{na}} \end{array}\right]=\;0 \end{array} \end{array}$$

By representing the expression in Equation (11) as **A** ***x*** = 0, the eigenmodes is determined from the singular decomposition of **A** given by **A** = USV*, where **S** contains the singular values and **V** contains the corresponding null space vectors, if any. The eigenmodes are determined using a numerical grid search, by searching for the local minima of the function min(**S**(*k*_x_)) in the complex *k*_x_ plane. The values of *k*_x_ are treated as eigenmodes (*k*_xn_) if the null space of **A** exists, which is determined using the condition min (**S**) < 10^−15^ norm(**A**). For each eigenmode, the complex coefficients are uniquely given by the null space vector corresponding to the smallest singular value. Since Equation (11) is satisfied for any scaling of the coefficients (*A*_na_,*B*_na_,*C*_na_,*D*_na_) and (*A*_nb_,*B*_nb_,*C*_nb_,*D*_nb_), the *y*‐component of the electric field in a grating layer is redefined as

(12) $$\begin{array}{c} \;E_{y}=\sum_{n}G_{n}\left(A_{n}e^{ik_{\mathrm{zn}}z}+B_{n}e^{-ik_{\mathrm{zn}}z}\right)e^{ik_{\mathrm{xn}}x}e^{i\left(k_{y}y-\omega t\right)}\; \end{array}$$

where *G*_n_ is a complex coefficient that weighs the various eigenmodes. This algorithm fully captures the well‐known eigenmodes of each grating layer, which can be verified by the continuity of the total electromagnetic fields across adjacent homogeneous and grating layers. The total electromagnetic fields are computed as the sum of all the diffracted and eigenmode waves with their complex coefficients determined by solving the scattering matrix (described in the next section), for which non‐trivial solutions does not exist if there are missing eigenmodes.

### Scattering Matrix Formulation

To determine the complex coefficients of each diffracted and eigenmode waves, the tangential electromagnetic fields is matched between adjacent layers, including the incident source field. In this work, the source field is the evanescent field of an electron in a homogeneous free space layer with incident wave‐vector *k*_zi_ = *ω*/*v*. Since the interface between layers lie in the *yz* plane, the electromagnetic fields *E*_y_, *E*_z_, *H*_y_, and *H_z_* must be continuous. The corresponding electromagnetic field components are then equated at each interface, e.g., the continuity of *E*_y_ between a homogeneous and grating layer is given by

(13) $$\begin{array}{c} \begin{array}{c} \sum_{m\;=\;-\infty }^{\infty }\left(a_{m}e^{ik_{\mathrm{xm}}x}\;+\;b_{m}e^{-ik_{\mathrm{xm}}x}\right)e^{ik_{\mathrm{zm}}z}\;=\;\sum_{n}G_{n}\left(A_{n}e^{ik_{\mathrm{zn}}z}\;+\;B_{n}e^{-ik_{\mathrm{zn}}z}\right)e^{ik_{\mathrm{xn}}x}\; \end{array} \end{array}$$

To simplify Equation (13), the Fourier series identity is used, given by

(14a) $$\begin{array}{c} f\left(z\right)=\sum_{m=-\infty }^{\infty }C_{m}\;e^{i\frac{2\pi m}{d}z} \end{array}$$

(14b) $$\begin{array}{c} \;C_{m}=\frac{1}{d}\;\int_{-\frac{rd}{2}}^{d-\frac{rd}{2}}f\left(z\right)e^{-i\frac{2\pi m}{d}z}dz \end{array}$$

which allow Equation (13) to be rewritten as

(15) $$\begin{array}{ccc} & & a_{m}e^{ik_{\mathrm{xm}}x}+b_{m}e^{-ik_{\mathrm{xm}}x}\\ & & \;=\sum_{n}G_{n}\left(A_{\mathrm{na}}\xi_{\mathrm{mna}+}+B_{\mathrm{na}}\xi_{\mathrm{mna}-}+A_{\mathrm{nb}}\xi_{\mathrm{mnb}+}+B_{\mathrm{nb}}\xi_{\mathrm{mnb}-}\right)e^{ik_{\mathrm{xn}}x} \end{array}$$

where

(16a) $$\begin{array}{c} \;\xi_{\mathrm{mna}\pm }=\;r\mathrm{sinc}\left[\frac{\left(\pm k_{\mathrm{zna}}-k_{\mathrm{zm}}\right)rd}{2}\right] \end{array}$$

(16b) $$\begin{array}{c} \;\xi_{\mathrm{mnb}\pm }=\left(1-r\right)\;e^{\frac{i\left(\pm k_{\mathrm{znb}}-k_{\mathrm{zm}}\right)d}{2}}\mathrm{sinc}\left[\frac{\left(\pm k_{\mathrm{znb}}-k_{\mathrm{zm}}\right)\left(1-r\right)d}{2}\right] \end{array}$$

with wave‐vectors $k_{\mathrm{zna}}={\left(k_{a}^{2}-k_{y}^{2}-k_{\mathrm{xn}}^{2}\right)}^{1/2}\;$ and $k_{\mathrm{znb}}={\left(k_{b}^{2}-k_{y}^{2}-k_{\mathrm{xn}}^{2}\right)}^{1/2}$. Note that one equation is given for each diffraction order and tangential field component at each interface. Therefore, the continuity of all four tangential electromagnetic fields for *M* number of diffraction modes and *L* layers results in a system of 4*M*(*L* − 1) linear equations, which is formulated into a scattering problem of the following form

(17) $$\left[\begin{array}{cccccccccc} \ddots & 0 & 0 & 0 & 0 & \vdots & \vdots & \ddots & \vdots & \ddots \\ 0 & \Gamma_{-1} & 0 & 0 & 0 & \Pi_{-1,\;1} & \Pi_{-1,\;2} & \ldots & \Pi_{-1,\;N} & \ddots \\ 0 & 0 & \Gamma_{0} & 0 & 0 & \Pi_{0,\;1} & \Pi_{0,\;2} & \ldots & \Pi_{0,\;N} & \ddots \\ 0 & 0 & 0 & \Gamma_{+1} & 0 & \Pi_{+1,\;1} & \Pi_{+1,\;2} & \ldots & \Pi_{+1,\;N} & \ddots \\ 0 & 0 & 0 & 0 & \ddots & \vdots & \vdots & \ddots & \vdots & \ddots \end{array}\right]\left[\begin{array}{c} \vdots \\ a_{m\;=\;-1}\\ b_{m\;=\;-1}\\ c_{m\;=\;-1}\\ d_{m\;=\;-1}\\ a_{m\;=\;0}\\ b_{m\;=\;0}\\ c_{m\;=\;0}\\ d_{m\;=\;0}\\ a_{m\;=\;+1}\\ b_{m\;=\;+1}\\ c_{m\;=\;+1}\\ d_{m\;=\;+1}\\ \vdots \\ G_{n\;=\;1}\\ G_{n\;=\;2}\\ \vdots \\ G_{n\;=\;N}\\ \vdots \end{array}\right]=\left[\begin{array}{c} \vdots \\ 0\\ 0\\ 0\\ 0\\ E_{\mathrm{yi}}\\ H_{\mathrm{zi}}\\ H_{\mathrm{yi}}\\ E_{\mathrm{zi}}\\ 0\\ 0\\ 0\\ 0\\ 0\\ 0\\ 0\\ 0\\ 0\\ \vdots \end{array}\right]$$

where *E*_yi_, *H*_zi_, *H*_yi_, and *E*_zi_ are the incident evanescent fields of the electron. The matrices **Γ**_**m**_ and column vectors **Π**_**m**,**n**_ are representations of the tangential electromagnetic fields (*E*_ym_,*H*_zm_,*H*_ym_,*E*_zm_) and (*E*_yn_,*H*_zn_,*H*_yn_,*E*_zn_) for the *m*th diffraction order and *n*th eigenmodes, respectively. Specifically, the matrix **Γ**_**m**_ is defined as

(18) $$\begin{array}{c} \;\Gamma_{m}=\Omega \left(\varepsilon ,\mu \right)\Lambda \left(k,k_{y}\right)K_{m}\left(k,k_{y},k_{\mathrm{zm}}\right)\Psi_{m}\left(k,k_{y},k_{\mathrm{zm}},\;x\right) \end{array}$$

where

(19a) $$K_{m}\left(k,k_{y},k_{\mathrm{zm}}\right)=\left[\begin{array}{cccc} 1 & 1 & 0 & 0\\ +kk_{\mathrm{xm}} & -kk_{\mathrm{xm}} & -k_{y}k_{\mathrm{zm}} & -k_{y}k_{\mathrm{zm}}\\ 0 & 0 & 1 & 1\\ -k_{y}k_{\mathrm{zm}} & -k_{y}k_{\mathrm{zm}} & -kk_{\mathrm{xm}} & +kk_{\mathrm{xm}} \end{array}\right]$$

(19b) $$\Psi_{m}\;\left(k,k_{y},k_{\mathrm{zm}},x\right)=\left[\begin{array}{cccc} e^{ik_{\mathrm{xm}}x} & 0 & 0 & 0\\ 0 & e^{-ik_{\mathrm{xm}}x} & 0 & 0\\ 0 & 0 & e^{ik_{\mathrm{xm}}x} & 0\\ 0 & 0 & 0 & e^{-ik_{\mathrm{xm}}x} \end{array}\right]$$

The vector **Π**_**m**,**n**_ is defined as

(20) $$\begin{array}{c} \;\Pi_{m,n}=\left[\begin{array}{cc} \Xi_{\mathrm{mna}} & \Xi_{\mathrm{mnb}\;} \end{array}\right]\;\left[\begin{array}{c} A_{\mathrm{na}}\\ B_{\mathrm{na}}\\ C_{\mathrm{na}}\\ D_{\mathrm{na}}\\ A_{\mathrm{nb}}\\ B_{\mathrm{nb}}\\ C_{\mathrm{nb}}\\ D_{\mathrm{nb}} \end{array}\right] \end{array}$$

where

(21a) $$\begin{array}{c} \Xi_{\mathrm{mna}}=\Omega \left(\varepsilon_{a},\mu_{a}\right)\Lambda \left(k_{a},k_{y}\right)K_{n}\left(k_{a},k_{y},k_{\mathrm{xn}}\right)\xi_{\mathrm{mna}}\left(k_{a},k_{y},k_{\mathrm{zm}},k_{\mathrm{xn}},\;r,\;d\right) \end{array}$$

(21b) $$\begin{array}{c} \Xi_{\mathrm{mnb}}=\;\Omega \left(\varepsilon_{b},\mu_{b}\right)\Lambda \left(k_{b},k_{y}\right)K_{n}\left(k_{b},k_{y},k_{\mathrm{xn}}\right)\xi_{\mathrm{mnb}}\left(k_{b},k_{y},k_{\mathrm{zm}},k_{\mathrm{xn}},\;r,\;d\right) \end{array}$$

(21c) $$\begin{array}{c} K_{n}\;\left(k,k_{y},k_{\mathrm{xn}}\right)=\left[\begin{array}{cccc} 1 & 1 & 0 & 0\\ +kk_{\mathrm{xn}} & +kk_{\mathrm{xn}} & -k_{y}k_{\mathrm{zn}} & +k_{y}k_{\mathrm{zn}}\\ 0 & 0 & 1 & 1\\ -k_{y}k_{\mathrm{zn}} & +k_{y}k_{\mathrm{zn}} & -kk_{\mathrm{xn}} & -kk_{\mathrm{xn}} \end{array}\right]\; \end{array}$$

(21d) $$\begin{array}{c} \xi_{\mathrm{mna}}\;\left(k_{a},k_{y},k_{\mathrm{zm}},\;k_{\mathrm{xn}},r,d\right)=\left[\begin{array}{cccc} \xi_{\mathrm{mna}+} & 0 & 0 & 0\\ 0 & \xi_{\mathrm{mna}-} & 0 & 0\\ 0 & 0 & \xi_{\mathrm{mna}+} & 0\\ 0 & 0 & 0 & \xi_{\mathrm{mna}-} \end{array}\right]\; \end{array}$$

(21e) $$\begin{array}{c} \xi_{\mathrm{mnb}}\;\left(k_{b},k_{y},k_{\mathrm{zm}},k_{\mathrm{xn}},\;r,d\right)=\left[\begin{array}{cccc} \xi_{\mathrm{mnb}+} & 0 & 0 & 0\\ 0 & \xi_{\mathrm{mnb}-} & 0 & 0\\ 0 & 0 & \xi_{\mathrm{mnb}+} & 0\\ 0 & 0 & 0 & \xi_{\mathrm{mnb}-} \end{array}\right]\; \end{array}$$

To determine the minimum number of modes required, it is noted that the first and last layer must be a homogeneous layer that extends to *x* = + ∞ and *x* = − ∞ respectively, with each layer having only 2 unknown coefficients as there are no waves propagating from infinity. Each homogeneous layer consists of 4*M* unknown coefficients (*a*_m_, *b*_m_, *c*_m_, and *d*_m_), while each grating layer contains *N* unknown coefficients (*G*_n_). Therefore, the total number of unknown coefficients for an arbitrary number of homogeneous layers (*L*_h_) and grating layers (*L*_g_) is 2*M* × (2) + 4*M* × (*L*_h_ − 2) + *N* × *L*_g_ which reduces to 4*M*(*L*_h_ − 1) + *NL*_g_ where *L* = *L*_h_ + *L*_g_. To solve the system of linear equations, the required number of equations must be equal to or greater than the number of unknown coefficients, given by the inequality 4*M*(*L* − 1) ≥ 4*M*(*L*_h_ − 1) + *NL*_g_, which simplifies to the condition *M* ≥ *N*/4. A preconditioning matrix (**P**) is also used to reduce the floating‐point errors in solving the system of linear equations in the form of **APP** ^−1^ ***x*** = **b**. The matrix **P** normalizes each column of matrix **A** to give normalized matrix **AP**, and the coefficients are solved by ***x*** = **P**((**AP**)^−1^ **b**). To ensure the convergence and accuracy of the computation, an intensity‐related quantity $I_{i}=\sum_{m}|c_{m}{|}^{2}\;$ is defined for the *i*th computation and the value of *N* is increased by factors of 2 for each new computation until the relative error Δ*I* = (*I*_i + 1_ − *I*_i_)/*I*_i + 1_ falls below 10^–6^.

### Electromagnetic Fields of Smith‐Purcell Radiation

Once the coefficients is determined from Equation (17), the total electromagnetic field of Smith‐Purcell radiation is computed by summing the propagating diffracted plane wave components across a desired range of frequencies (*ω*) and wave‐vectors (*k*_y_). A frequency filter with transmission spectrum *f*(*ω*) is also applied to the coefficients, which in this case is a rectangular filter with no phase shift:

(22) $$f\left(\omega \right)=\left\{\begin{array}{cc} 1, & \omega_{\min}<\omega <\omega_{\max}\\ 0, & \mathrm{otherwise} \end{array}\right.$$

where Δ*ω* = *ω*_max_ − *ω*_min_ is the frequency filter bandwidth. The resulting *y*‐component electric field of the filtered Smith‐Purcell radiation is given by

(23) $$\begin{array}{c} \;E_{y}=\int \;\;\;\int f\left(\omega \right)\sum_{m}\left(a_{m}e^{ik_{\mathrm{xm}}x}+b_{m}e^{-ik_{\mathrm{xm}}x}\right)e^{ik_{\mathrm{zm}}z}e^{i\left(k_{y}y-\omega t\right)}\;dk_{y}\;d\omega \end{array}$$

The total Smith‐Purcell radiation from an electron bunch is then obtained by assuming a linear superposition of phase‐compensated Smith‐Purcell radiation from individual electrons. Using the simulated results of Smith‐Purcell radiation generated by a single electron positioned at (*x*_0_,*y*_0_, *z*_0_), the electromagnetic fields generated by other electrons of the same velocity at different position (*x*_s_,*y*_s_,*z*_s_) is obtained by including an additional phase factor

(24) $$\begin{array}{c} \;\psi_{\mathrm{ms}}=e^{ik_{x}\left(x_{s}-x_{0}\right)}\;e^{-i\left(k_{\mathrm{zm}}\left(z_{s}-z_{0}\right)+k_{y}\left(y_{s}-y_{0}\right)\right)} \end{array}$$

where the term $e^{ik_{x}\left(x_{s}-x_{0}\right)}$ accounts for the change in incident evanescent field strength, and the term $e^{-i(k_{\mathrm{zm}}\left(z_{s}-z_{0}\right)+k_{y}\left(y_{s}-y_{0}\right))}$accounts for the phase shift of the diffracted waves. The *y*‐component electric field of the filtered Smith‐Purcell radiation from an electron bunch is then calculated as

(25) $$\begin{array}{c} \begin{array}{c} E_{y}=\int \;\;\;\int f\left(\omega \right)\sum_{m}\sum_{s}\psi_{\mathrm{ms}}\left(a_{m}e^{ik_{\mathrm{xm}}x}+b_{m}e^{-ik_{\mathrm{xm}}x}\right)e^{ik_{\mathrm{zm}}z}e^{i\left(k_{y}y-\omega t\right)}\;\;dk_{y}\;d\omega \end{array} \end{array}$$

The other Cartesian components of electric fields are obtained from Equations (4a) and (4b), and the magnetic fields are obtained using the exchange operation ***E*** ↔ ***H*** and + *ε*↔ − *μ*.

Note that in this study, the electromagnetic response of a non‐magnetic grating in free space (*μ*_a_ = *μ*_0_, *ε*_b_ = *ε*_0_, and *μ*_b_ = *μ*_0_) is considered, which corresponds to the specialized case of a single grating layer between two homogeneous layers.

## Conflict of Interest

The authors declare no conflict of interest.

## Supporting information

Supporting Information

## Data Availability

The data that support the findings of this study are available from the corresponding author upon reasonable request.
