# Supplementary material for: Space‐Time Wave Packets from Smith‐Purcell Radiation
Source: Adv Sci (Weinh). 2021 Oct 17;8(22):2100925. doi: 10.1002/advs.202100925 (PMC8596120; doi:10.1002/advs.202100925)
Supplement: Supplementary file 1 — Supporting Information [file ADVS-8-2100925-s001.pdf]

ADVANCED SCIENCE
Open Access

Supporting Information

for *Adv. Sci.*, DOI: 10.1002/advs.202100925

Space-time wave packets from Smith-Purcell radiation

*Yi Ji Tan, Prakash Pitchappa, Nan Wang, Ranjan Singh and Liang Jie Wong**

Supporting Information

Space-time wave packets from Smith-Purcell radiation

*Yi Ji Tan, Prakash Pitchappa, Nan Wang, Ranjan Singh and Liang Jie Wong**

S1. Effects of space charge on the divergence of electron bunches

The electromagnetic fields of an electron in uniform motion remains propagation-invariant in the absence of external forces. However, space charge effects – i.e., the electromagnetic repulsion between electrons – leads to an increase in the transverse velocity of electron bunches. As such, an electron bunch is propagation-invariant only as far as space charge effects remains negligible. We model space charge by considering the electromagnetic forces acting on each electron, given by the Newton-Lorentz equation

$$\frac{d\boldsymbol{p}}{dt} = q(\boldsymbol{E} + \boldsymbol{v} \times \boldsymbol{B}), \tag{S1}$$

where q and m are the electron's charge and mass respectively, relativistic momentum $\boldsymbol{p} = \gamma m\boldsymbol{v}$, $\boldsymbol{E}$ and $\boldsymbol{B}$ are the electromagnetic fields acting on the electron, Lorentz factor $\gamma = (1 - v^2/c^2)^{-1/2}$, $\boldsymbol{v}$ is the velocity vector, and velocity $v = |\boldsymbol{v}|$. We rewrite the velocity as a function of momentum:

$$\boldsymbol{v} = \frac{d\boldsymbol{r}}{dt} = \frac{\boldsymbol{p}c}{\sqrt{p^2 + m^2c^2}} \tag{S2}$$

where $\boldsymbol{r}$ is position vector, and momentum $p = |\boldsymbol{p}|$. We calculate the total electromagnetic fields acting on each electron as the sum of electromagnetic fields from all the other electrons in a bunch. The electromagnetic fields of an electron are derived from the Liénard–Wiechert potentials, which models each electron as a point charge. The Newton-Lorentz equations allow us to obtain the trajectory of each electron, which we calculate by numerically integrating Equation S1 and S2 using the Runge-Kutta formulas.[1] At each time step of the numerical

integration, we assume each electron to be propagating at a constant velocity with electromagnetic fields given by

$$\boldsymbol{E}(\boldsymbol{r_p}) = \frac{q}{4\pi\varepsilon_0}\frac{1 - v^2/c^2}{\left(1 - \frac{v^2}{c^2}\sin^2\alpha\right)^{3/2}}\frac{\boldsymbol{r_p}}{r_p^3}, \tag{S3a}$$

$$\boldsymbol{B}(\boldsymbol{r_p}) = \frac{1}{c^2}\left(\boldsymbol{v} \times \boldsymbol{E}(\boldsymbol{r_p})\right), \tag{S3b}$$

where ε_0 is the permittivity of free space, $\boldsymbol{r_p}$ is the position vector pointing from the electron's position, and α is the angle between vectors $\boldsymbol{r_p}$ and $\boldsymbol{v}$.

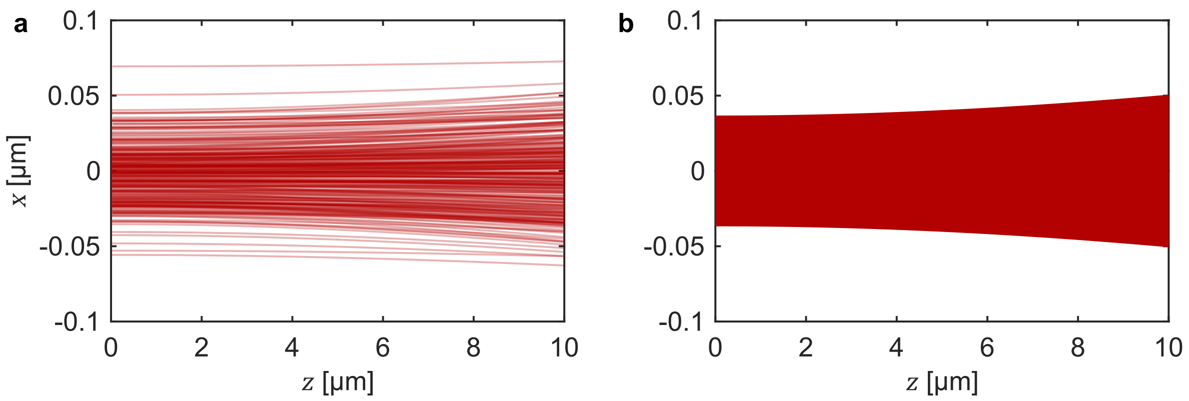

Figure S1. Divergence of 200 keV electrons. a) Trajectory of 250 electrons normally distributed in a spherical bunch of full-width-at-half-maximum (FWHM) diameter 50 nm, calculated by numerically integrating the Newton-Lorentz equations using the Runge-Kutta formulas. b) Divergence of a uniformly distributed circular beam of current 83.5 mA, calculated using an analytical model of circular beam.

Figure S1a shows the trajectory of a 200 keV electron bunch containing 250 electrons normally distributed with a 50 nm FWHM diameter, calculated by numerically integrating the Newton-Lorentz equations. We also calculate the divergence of an uniformly distributed circular beam in Figure S1b, using an analytical model of space charge,[2-4] which describes the propagation distance (z) and beam divergence (θ_d) as

$$z = \frac{2R_0}{\sqrt{qI/\pi m\varepsilon_0\gamma^3v^3}}\int_0^{\sqrt{\ln(R/R_0)}} e^{t^2}\,dt, \tag{S4a}$$

$$\theta_d = \arctan\left(\frac{dR}{dz}\right) = \arctan\left(\sqrt{\frac{qI}{\pi m\varepsilon_0\gamma^3v^3}\ln\left(\frac{R}{R_0}\right)}\right), \tag{S4b}$$

where R_0 is the beam radius at $z = 0$, R is the beam radius, and I is the beam current.

The standard deviation of a uniformly distributed circular beam is given by $\sigma = \sqrt{1/3}R_0$, whereas the FWHM diameter of a normally distributed spherical bunch is related to the standard deviation by $d_{\mathrm{FWHM}} = 2\sqrt{2\ln 2}\,\sigma$. The standard deviation of the two distributions allows us to obtain an equivalent beam radius of a spherical bunch given by $R_0 = d_{\mathrm{FWHM}}/\sqrt{(8/3)\ln 2}$, where d_{FWHM} is the FWHM diameter. We also define an equivalent beam current $I = Nqv/(2d_{\mathrm{FWHM}})$, where N is the total number of electrons. A 0.04 fC, 200 keV electron bunch of FWHM diameter 50 nm is thus equivalent to a circular beam of current 83.5 mA. Equation S4b also allows us to calculate the transverse velocity as

$$v_{\perp} = v\frac{dR}{dz} = v\sqrt{\frac{qI}{\pi m\varepsilon_0\gamma^3 v^3}\ln\left(\frac{R}{R_0}\right)}. \tag{S5}$$

We assume a normal distribution for the transverse velocity and define the transverse velocity spread as $\sigma(v_{\perp}) = |v_{\perp}|/2$ for the analytical model. For the circular beam of current 83.5 mA shown in Figure S1b, we predict a normalized transverse velocity spread $\sigma(v_{\perp}/v)$ = 0.07% at propagation distance z = 5 μm. Our space charge calculation also predicts a 0.07% normalized transverse velocity spread at z = 5 μm and a 0.16% energy spread for the electron bunch in Figure S1a. These parameters translate to a 0.04 fC, 200 keV electron bunch of FWHM diameter 50 nm that is reasonably propagation-invariant over a 10 μm distance (from $z = -5$ μm to $+5$ μm). Longer propagation distances can be realized by decreasing the electron density, and by using relativistic electrons of higher energy.

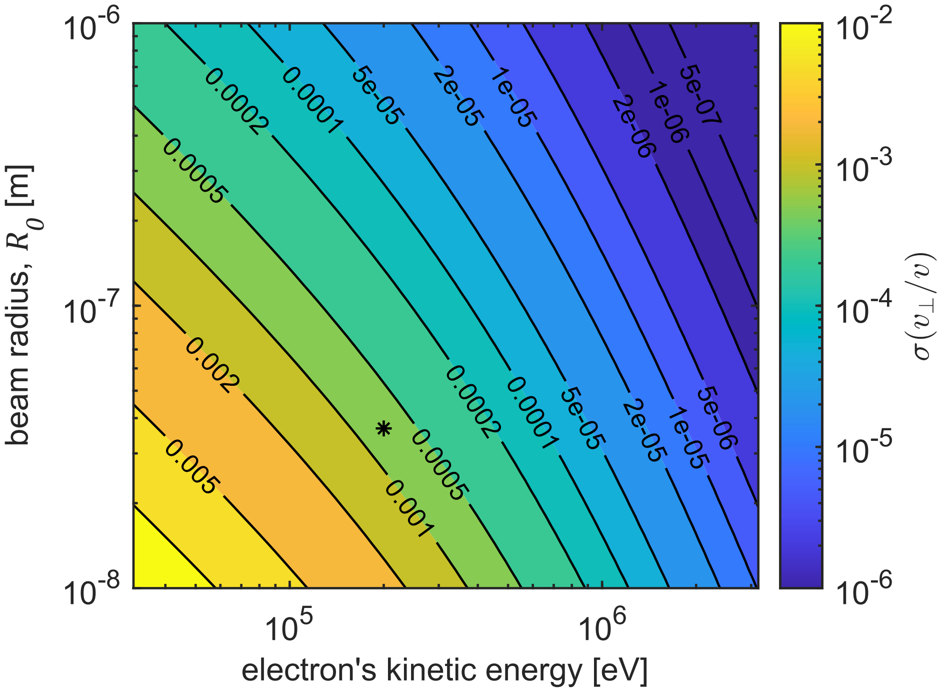

Figure S2. Normalized transverse velocity spread of electron bunches for different beam radius and electron energies. The colormap shows the normalized transverse velocity spread of circular beams (with current equivalent to 250 electrons in a spherical bunch) as a function of electron energy and beam radius R_0, at propagation distance $z = 100\ R_0$. The point corresponding to a 200 keV electron bunch of FWHM diameter 50 nm is marked by an asterisk.

In **Figure S2**, we calculate the normalized transverse velocity spread of circular beams (with current equivalent to 250 electrons in a spherical bunch) as a function of electron energy and beam radius. Our calculations show that the normalized transverse velocity spread is relatively small for electron beams with high energy and large beam radius. For comparison, we mark the point corresponding to a 200 keV electron bunch of FWHM diameter 50 nm with an asterisk.

Supporting References

[1] J. R. Dormand, P. J. Prince, *J. Comput. Appl. Math.* **1980**, *6*, 19.

[2] L. J. Wong, I. Kaminer, O. Ilic, J. D. Joannopoulos, M. Soljačić, *Nat. Photonics* **2016**, *10*, 46.

[3] R. Pakter, C. Chen, *Phys. Rev. E* **2000**, *62*, 2789.

[4] M. Reiser, *Theory and design of charged particle beams*, Wiley, New York, **1994**.
